# Evaluation of RNA isolation methods for microRNA quantification in a range of clinical biofluids

**DOI:** 10.1186/s12896-021-00706-6

**Published:** 2021-08-06

**Authors:** Henk P. Roest, Jan N. M. IJzermans, Luc J. W. van der Laan

**Affiliations:** Department of Surgery, Laboratory of Experimental Transplantation and Intestinal Surgery (LETIS), Erasmus MC – University Medical Center, P.O. Box 2040, Room Na-1005, 3000 CA Rotterdam, the Netherlands

**Keywords:** microRNA, RT-qPCR, Biofluid, miRNA isolation, Heparin contamination, Graft preservation

## Abstract

**Background:**

Extracellular microRNAs (miRNAs), released from cells into biofluids, have emerged as promising biomarkers for diagnostic and prognostic purposes. Several RNA isolation methods are available for the analysis of these cell-free miRNAs by RT-qPCR. Not all methods, however, are equally suitable for different biofluids. Here, we extracted total RNA from four very diverse biofluids: serum, urine, bile, and graft preservation fluid (perfusate). Four different protocols were used: a phenol-chloroform extraction and alcohol precipitation in combination with a precipitation carrier (QP) and three different column-based isolation methods, one with phenol-chloroform extraction (RN) and two without (NG and CU). For this range of clinical biofluid samples, we evaluated the potential of these different RNA isolation methods assessing recovery efficiency and the co-purification of RT-qPCR inhibiting compounds.

**Results:**

Differences were observed between each of the RNA isolation methods in the recovery of cel-miR-39, a synthetic miRNA spiked in during the workup procedure, and for endogenous miRNAs. Co-purification of heparin, a known RT-qPCR inhibitor, was assessed using heparinase I during cDNA synthesis. RT-qPCR detection of synthetic miRNAs cel-miR-39, spiked in during RNA workup, cel-miR-54, spiked in during cDNA synthesis, and endogenous miRNAs was strongly improved in the presence of heparinase I for some, but not all, isolation methods. Other, co-isolated RT-qPCR inhibitors were not identified, except for biliverdin, which co-isolated from some bile samples with one of the methods. In addition, we observed that serum and urine contain compounds that enhance the binding of heparin to certain solid-phase columns.

**Conclusions:**

For reliable measurements of miRNA-based biomarkers in biofluids, optimization of RNA isolation procedures is recommended as methods can differ in miRNA detection and in co-purification of RT-qPCR inhibitory compounds. Heparinase I treatment confirmed that heparin appeared to be the major RT-qPCR inhibiting compound, but also biliverdin, co-isolated from bile, could interfere with detection.

**Supplementary Information:**

The online version contains supplementary material available at 10.1186/s12896-021-00706-6.

## Background

Small, non-coding RNAs have been identified in both prokaryotic and eukaryotic organisms where they participate in a wide range of regulatory events [1]. MicroRNAs (miRNAs) are a family of non-coding RNAs of approximately 18–24 nucleotides in length that are essential for post-transcriptional regulation of gene expression. With an estimated 60% of all genes being post-transcriptionally regulated by these molecules, miRNAs play an important role in many fundamental, biological processes [2, 3]. Over 2500 different human miRNAs have been deposited in publicly available databases like miRBase [4], but it is likely that new ones will still be identified [5]. Observations that miRNA profiles are frequently altered during cellular development and pathology indicate an important role in both malignant and non-malignant diseases [6]. In addition, many miRNAs are also expressed in a tissue- or organ-specific manner, suggesting that miRNAs likely have high specificity and are applicable as biomarkers.

The discovery of disease-related variations of miRNAs in blood or urine, for instance, highlights their potential as minimal or non-invasive biomarkers [7]. A plethora of studies were initiated, in which cell-free miRNAs in serum or plasma were explored as biomarkers for disease diagnosis, prognosis and monitoring of treatment responses. Although initially explored in cancer [8], changes in serum/plasma miRNA composition have also been observed for many other diseases, including cardiovascular, neurodegenerative and liver diseases [9–12]. As extracellular miRNAs can reflect disease progression and treatment effects, they can also reflect tissue injury and graft outcome in the setting of organ transplantation [9, 13–16]. Subsequent studies identified miRNAs in virtually all body fluids including breast milk, saliva, urine, cerebrospinal fluid, semen and bile, as well as some non-bodily biofluids like organ preservation fluid (perfusate), broncho-alveolar lavage fluid and peritoneal dialysis effluent [17–22].

Two important properties of potential biomarkers are (i) ease of attainment of clinical samples, and (ii) robustness and ease of the detection assay. With the discovery of miRNAs and the presence of these molecules in biofluids, the condition for ease of attainment has been readily solved. Robustness of miRNA assays is challenged at multiple levels, as it requires stability during sampling, storage, and subsequent processing, and must also be executed in a consistent and reproducible fashion with as little technical variation as possible [23]. Whereas stability of miRNAs during sampling and storage has been confirmed [21, 24–27], and detection using validated commercial assays is widely available, the optimal isolation procedure for miRNAs from different biofluids remains less established. Several studies have been published which address the question of miRNA isolation [28–31], but often yield inconsistent or sometimes even controversial results, and do not consider the presence of co-purified inhibitory compounds like heparin [32, 33]. Many methods and commercial kits are available for miRNA isolation of biofluids, but data on which method/kit is most suitable for which biofluid is still lacking. Therefore, the aim of this study is to determine the most robust and all-round miRNA isolation procedure for a number of human biofluids obtained in clinical settings.

## Results

### Evaluation of miRNA recovery during isolation for different biofluids

**Fig. 1:**
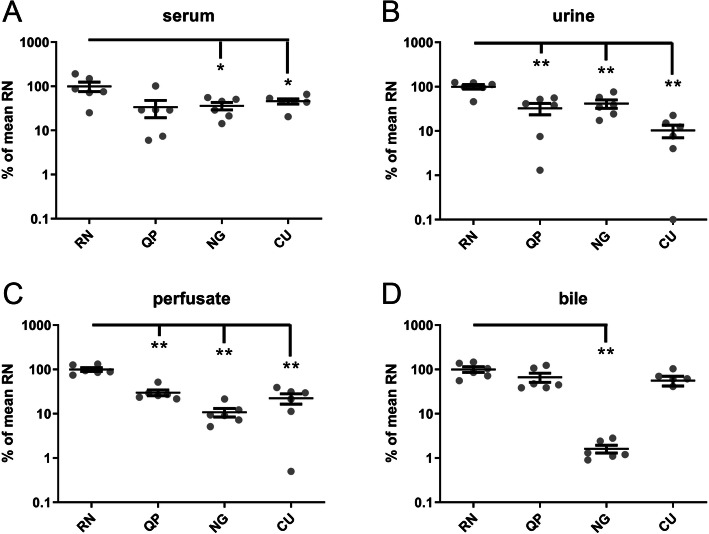
Relative detection levels of spiked-in cel-miR-39 in four biofluids. Levels of cel-miR-39, spiked during workup, were determined in serum (**A**), urine (**B**), perfusate (**C**), and bile (**D**) from six patients and their donor organs. Results are shown of four different methods and presented as scatter plots (mean ± SEM). RN; Qiagen miRNeasy kit, QP; Qiazol in combination with the dr. GenTLE precipitation carrier, NG; NORGEN Total RNA isolation kit, and CU; miRCURY RNA isolation kit - biofluids. The mean of the levels with the RN isolation method per liquid were set at 100% . Data are presented as mean ± SEM. *: *p* < 0.05, **: *p* < 0.01, Mann-Whitney U test

The robustness of an assay is an essential parameter for miRNA detection. To test the miRNA recovery, the levels of a synthetic spiked-in miRNA (cel-miR-39) in six matched human serum, urine, bile and perfusate samples were measured using the four aforementioned methods. The direct output of the PCR results showed that the median Cq values were the lowest for the RN method in all 4 analyzed biofluids (23.15, 23.26, 22.3, and 22.85 for serum, urine, perfusate, and bile, respectively) and the most consistent, as determined by the smallest range, in urine, perfusate, and bile (23.01-24.45, 21.76-22.59, and 22.21-23.62, respectively). To determine the relative improvement in detection using the RN method in comparison with the QP, NG and CU methods, relative detection levels were converted to percentages with the mean of the RN method for each biofluid set to 100% (Fig. 1). The mean recovery of cel-miR-39 from serum using the RN method was significantly better than the recovery using the QP and CU method with levels (mean% ± SEM) reaching only 33.8% ± 14.4 and 45.8% ± 6.1 of the levels of the RN method, respectively. Recovery from serum using the NG method was also less than half of the RN method (36.1% ± 6.9), but not significantly different from RN (Fig. 1A). The recovery improvement of cel-miR-39 was even more pronounced in RNA isolated from urine samples. The RN method performed significantly better than methods QP, NG, and CU, with recovery levels of 32.5% ± 9.3, 41.5% ± 8.9, and 10.3% ± 3.3, respectively when compared with the RN method (Fig. 1B). The significantly better performance of the RN method was also observed for RNA isolated from perfusate with recovery levels reaching only 29.8% ± 4.4, 10.8% ± 2.3, and 22.2% ± 5.8 for QP, NG, and CU, respectively, compared with the RN method (Fig. 1C). Although recovery of RNA from bile was better with method RN, the levels were not significantly improved when compared with the QP and CU method (66.6% ± 15.5 and 56.1% ± 14.0). Recovery from bile using the NG method, however, was significantly lower (1.6% ± 0.3) (Fig. 1D).

**Fig. 2:**
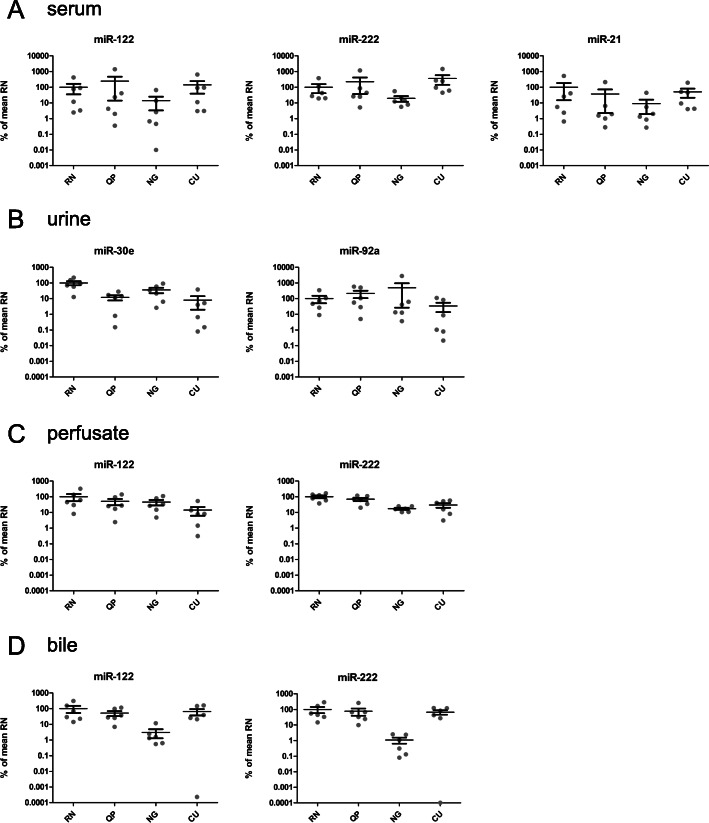
Quantification of endogenous miRNA recovery in four biofluids. **A**) Relative detection levels of endogenous human miRNAs miR-122, miR-222 and miR-21 in serum, **B**) miR-30a and miR-92e in urine, **C**) miR-122 and miR-222 in perfusate, and **D**) miR-122 and miR-222 in bile. The mean of the levels with the RN isolation method per individual miRNA per liquid were set at 100%. Bars represent the mean ± SEM of the six individual samples. RN; Qiagen miRNeasy kit, QP; Qiazol in combination with the dr. GenTLE precipitation carrier, NG; NORGEN Total RNA isolation kit, and CU; miRCURY RNA isolation kit - biofluids

The effect of the different RNA isolation methods on the yield of endogenous miRNA present in the four biofluids is shown in Fig. 2. For serum, miR-122, miR-222 and miR-21 were measured (Fig. 2A). For urine, miR-30a and miR-92e were determined (Fig. 2B), for the biofluids bile and PF both miR-122 and miR-222 were measured (Fig. 2C and D). The overall trend in detection levels of the different RNA isolation methods within one biofluid of the endogenous miRNAs is similar to that of the spiked-in miRNA cel-miR-39. Some clear differences, however, are also observed. Detection in RNA from serum after isolation with method CU was comparable to detection in RNA isolated with method RN (Fig. 2A).

### Isolation methods differ in the co-purification of heparin

**Fig. 3:**
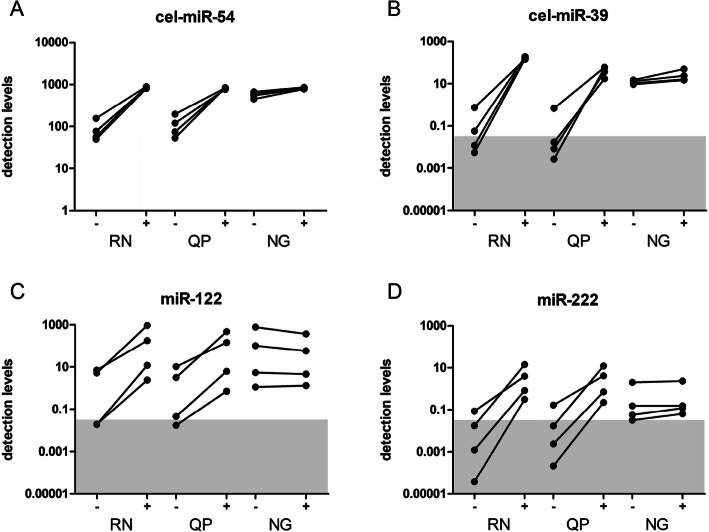
Heparinase I treatment improves quantification levels in biofluids with high heparin contamination. Total RNA from four different graft perfusate samples known to contain heparin were co-incubated with (+) or without (−) 6 IU heparinase I during cDNA synthesis. Results are shown for spike in miRNAs cel-miR-54 (**A**), cel-miR-39 (**B**) and endogenous miRNAs, miR-122 (**C**) and miR-222 (**D**) for three different RNA isolated methods, RN; Qiagen miRNeasy kit, QP; Qiazol in combination with the dr. GenTLE precipitation carrier, NG; NORGEN Total RNA isolation kit. Notably the NG method is the least sensitive for heparin co-purification and showed the smallest differences in detection levels between with or without heparinase I. Detection levels are calculated from Cq values using the following equation: detection levels = 2^(−Cq)^× 10^9^

As shown in multiple studies, anti-coagulants can strongly affect the analysis of miRNAs for biomarker discovery. Heparin contamination is known to negatively influence the quantification of miRNAs in blood samples and urine, as well as in perfusate. To circumvent this specific inhibition, the use of heparinase I in cDNA synthesis was propagated [34–36]. To assess any possible differences in co-isolation of heparin between the different methods, four perfusate samples with proven heparin contamination were selected from a previous study [36]. Total RNA was isolated using methods QP, RN, and NG, and RT-qPCR was performed in the absence (−) or presence (+) of heparinase I. The RT-qPCR results for cel-miR-54 in the presence and absence of heparinase I showed a clear increase in relative detection levels for methods RN and QP, while the improvement with RNA isolated with the NG method is only marginal (Fig. 3A). Results for recovery of cel-miR-39 were presented as relative detection levels in Fig. 3B. Co-purification of heparin was most prominent in RNA isolated using methods QP and RN, where treatment with heparinase I resulted in a mean increase of the relative detection levels of 804-fold and 236-fold, respectively. Heparinase I treatment during cDNA synthesis of RNA isolated using the NG method, on the other hand, only resulted in a 2-fold improvement (Fig. 3B). As already shown previously, relative detection levels after heparinase I treatment were the highest in RNA isolated using method RN. We also tested the effect of heparin contamination on endogenous miRNAs miR-122 and miR-222, two miRNAs that have shown to be well detectable in perfusate samples [17]. Again, detection of miRNAs clearly improved when heparinase I treatment was applied to RNA isolated through methods QP and RN. RNA isolated with method NG, again, showed little, if any, evidence of heparin co-purification as no improvement was observed after treatment with heparinase I for miR-122 and miR-222 (Fig. 3C and D). The RT-qPCR results for cel-miR-54 in the presence of heparinase I also showed that no additional inhibitory compounds, significantly contributing to a decrease in detection levels, were isolated that are specific to one of these 3 methods as mean relative detection levels after heparinase I treatment for methods RN, QP, and NG were 856, 820, and 825, respectively (Fig. 3A).

### The RNA isolation procedure NG is not completely insensitive to heparin contamination

**Fig. 4:**
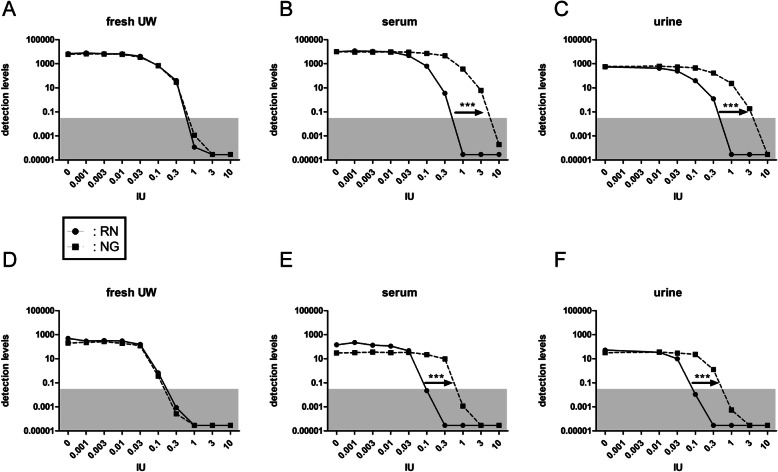
Differences in heparin sensitivity between isolation methods is only observed in body fluids. Biofluids were spiked with increasing amounts of unfractionated heparin. Ten μl of a half-log dilution series containing the indicated IU of heparin were added to the samples prior to RNA isolation. Cel-miR-39 for loss during work-up (**A, B, C**) and cel-miR-54 as indicator of co-purified RT-qPCR inhibiting compounds (**D, E, F**) were determined in RNA from preservation fluid (fresh UW) (**A, D**), or from the body fluids serum (**B, E**), and urine (**C, F**), using method RN (miRNEAsy kit, black circles/solid lines) and method NG (Norgen kit, black squares/dotted lines). Heparin amounts are shown on the x-axis. The area of unreliable detection is indicated in grey. Detection levels are calculated from Cq values using the following equation: detection levels = 2^(−Cq)^× 10^9^. In serum and urine, the inhibitory effect of heparin is approximately ten times higher with the RN method compared to NG method as indicated by arrows. No difference was observed for both spiked-in cel-miR-39 and cel-miR-54 isolated from fresh UW using method NG or RN. ***: *p* < 0.001, nonlinear regression (curve fit) comparison for dose-response data

Initially considered as a method that could isolate RNA without heparin contamination, results suggested that a certain level of heparin contamination could still be detected in the RNA samples isolated with the NG method in the absence of heparinase I (Fig. 3). To follow up on this observation and to determine to what extend and to what level heparin contamination is present in RNA samples isolated with the NG method, clean (un-used) UW solution was spiked with the standard amount of cel-miR-39 (200 amol) in combination with increasing amounts of heparin. RNA was isolated using either the NG or the RN method, and RT-qPCR detection of cel-miR-39 and cel-miR-54 - the latter added during cDNA synthesis - was performed in the absence of heparinase I. For both cel-miR-54 (Fig. 4A) and cel-miR-39 (Fig. 4D), the reduction of detection levels with increasing amounts of heparin was very similar between methods RN and NG, suggesting that the latter procedure does not prevent heparin contamination. To determine whether the observed discrepancy was caused by components only present in body fluids, urine and serum samples from four patients were pooled and also spiked with increasing amounts of heparin. Spike-in miRNAs cel-miR-39 and cel-miR-54 were determined as described for the clean UW samples. Nonlinear regression comparison (i.e. curve fitting) of dose-response data on log-transformed, normalized duplicates was used for statistical analysis. RNA isolated with the NG method from both serum and urine appeared significantly less contaminated with heparin when compared to RNA isolated with the RN method (*p* < 0.0001). This was determined for both for the spike-in added during the work-up procedure (Fig. 4B and C) as well as for the spike-in added during the cDNA synthesis procedure (Fig. 4E and F). This indicated the presence of components in body fluids that prevent co-isolation of heparin with the NG method.

### Evaluation of co-purification of PCR-inhibiting compounds other than heparin

**Fig. 5:**
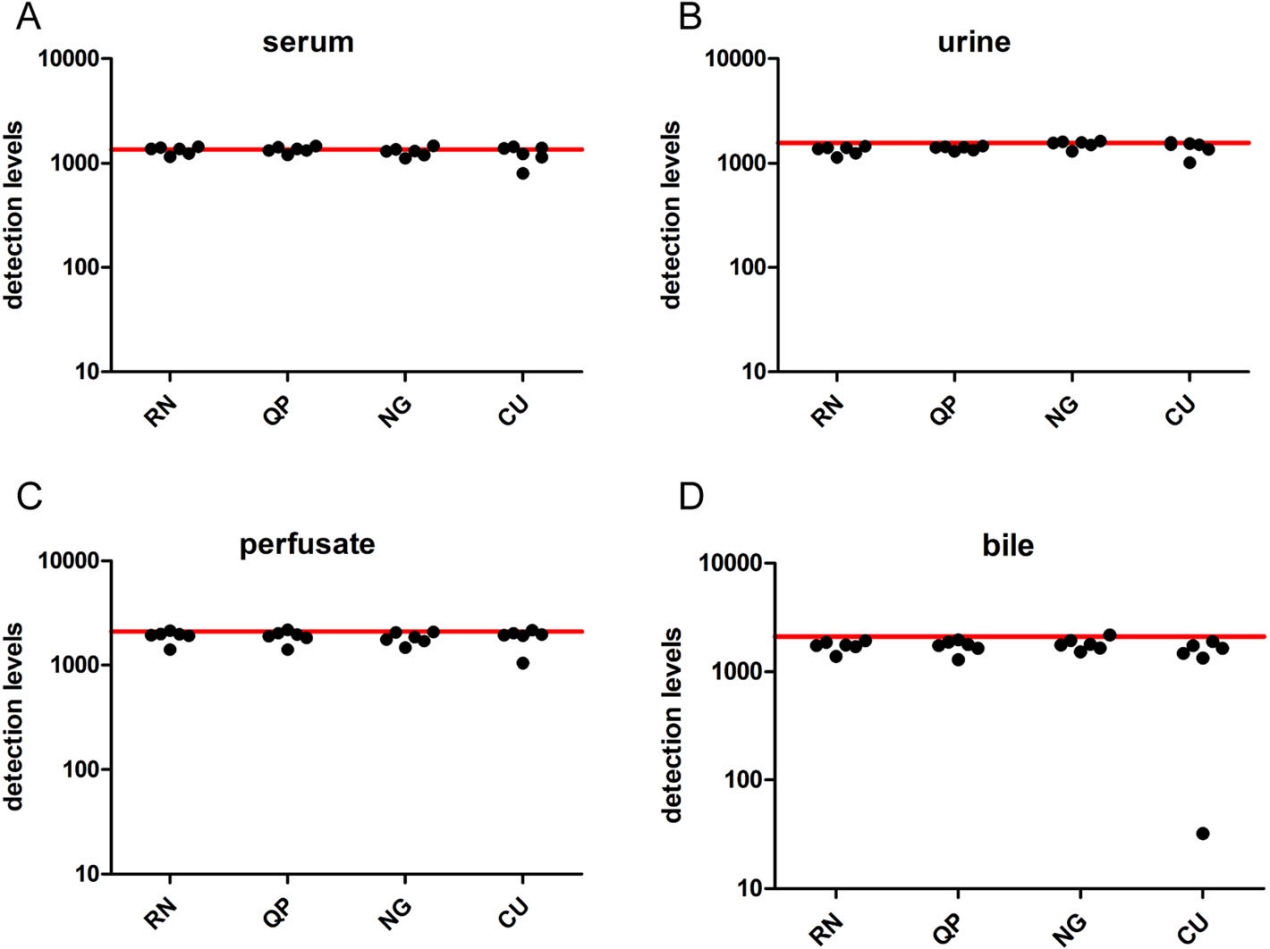
Detection levels of spiked-in cel-miR-54 in serum,urine, perfusate and bile. Levels of cel-miR-54, spiked during cDNA synthesis, are determined in serum (**A**), urine (**B**), perfusate (**C**), and bile (**D**) from six liver patients and their donor organs. All samples were treated with Heparinase I, and no statistically significant differences were observed between groups. Red lines indicate the detection levels of the reference sample (cel-miR-54 spiked in RNA-free water). RN; Qiagen miRNeasy kit, QP; Qiazol in combination with the dr. GenTLE precipitation carrier, NG; NORGEN Total RNA isolation kit, and CU; miRCURY RNA isolation kit – biofluids. Detection levels are calculated from Cq values using the following equation: detection levels = 2^(−Cq)^× 10^9^

RT-qPCR analyses are prone to interference by compounds that co-purify with RNA [37]. Besides heparin, other interfering compounds can also be co-purified which cannot be counteracted by heparinase I treatment. As already shown, a substantial variation was observed in the detection levels of cel-miR-39, hinting at the presence of non-heparinous compounds. Therefore the RNA samples used previously (obtained from serum, urine, perfusate and bile of six patients), were spiked with cel-miR-54, and, in the presence of heparinase I, cDNA was synthesized, followed by RT-qPCR analysis. Detection levels of cel-miR-54 in serum were not significantly different between methods RN, QP, NG, and CU, with values ranging between 1157-1444, 1207-1465, 1122-1473 and 802-1441,respectively (Figure 5A). Results for urine were comparable, with respective values ranging from 1140-1457, 1310-1468, 1309-1637 and 1012-1591 for methods RN, QP, NG, and CU (Figure 5B). Results for perfusate were also not different between the four different methods with values ranging between 1408-2141, 1406-2181, 1471-2082 and 1049-2167 for RN, QP, NG and CU, respectively (Figure 5C). As the reference values of cel-miR-54 - indicated by the red line in Figure 5 - were 1358, 1571 and 2101 for serum, urine and perfusate, respectively, the co-purification of other PCR-inhibiting compounds from these biofluids seemed almost absent.

**Fig. 6:**
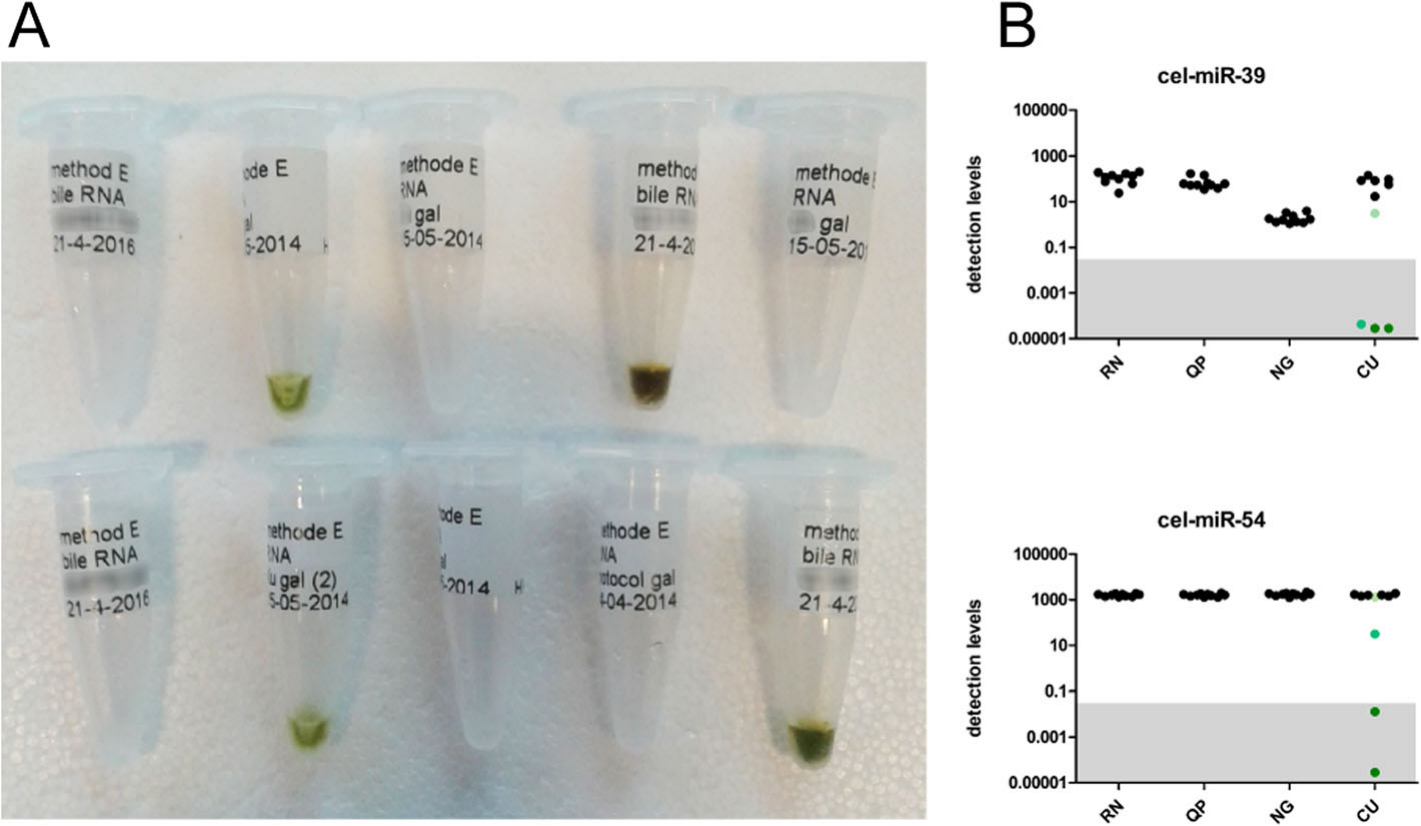
Colorimetric effects on RT-qPCR results in bile. (**A**) Visual observation of total RNA isolated from ten bile samples using the miRCURY RNA isolation kit – biofluids showed four samples had greenish discoloration. RNA samples isolated by other methods did not show any coloration (not shown) (**B**) The coloration of RNA samples correlated with low detection levels of cel-miR-39 (upper panel) and cel-miR-54 (lower panel). Black circles represent non-colored samples, green circles represent the greenish discolored samples. The unreliable detection area is indicated in grey. Detection levels are calculated from Cq values using the following equation: detection levels = 2^(−Cq)^× 10^9^. RN; Qiagen miRNeasy kit, QP; Qiazol in combination with dr. GenTLE precipitation carrier, NG; NORGEN Total RNA isolation kit, CU; miRCURY RNA isolation kit - biofluids

Contrary to serum, urine and perfusate, levels of spike-in cel-miR-54 in RNA samples from bile showed a larger extent of variation. Detection levels range from 1373 to 1917, 1288–1958, and 1515–2165 for the RN, QP, and NG method, but 32–1884 for the CU method (Fig. 5D). This increased range for cel-miR-54 levels in RNA from bile could be attributed to one sample that showed a distinct green discoloration. To follow up on this observation, the number of bile samples analyzed was increased to 10, and cel-miR-39 and cel-miR-54 levels were again determined. Four out of 10 RNA samples showed this discoloration after isolation (Fig. 6A), and this phenomenon was associated with lower detection levels of both cel-miR-39 and cel-miR-54, which was most likely caused by co-isolation of biliverdin when the CU method was used (Fig. 6B).

## Discussion

In this study, we evaluated the efficiency of four different methods to isolate miRNAs from some very unique and diverse biofluids, explored their use in more common biofluids like serum and urine, and determined their sensitivity towards contamination by the anti-coagulant heparin, a clinically relevant and frequently used drug. To our knowledge, this is the first report that describes a systematic approach to determine the most optimal method for a range of biofluids. In addition to the set of specific biofluids (bile and perfusate), we also determined their applicability of the different methods on biofluids more relevant for biomarker discovery and measurement, and in that way, identify the most robust method for general use. Many different methods and kits have been developed that allow for the isolation of total RNA without the loss of small RNA. From our results, we concluded that the miRNeasy kit was the most effective in isolating small RNA molecules from two very distinct biofluids, bile and perfusate, as measured by RT-qPCR, while retaining qualitatively and quantitatively good levels of detection in serum and urine.

Analyzing the results for each individual method, the recovery of cel-miR-39 displayed a comparable pattern as we observed with the endogenous miRNAs (Figs. 1 and 2). Identification of robust controls for data normalization remains a challenge when biofluids are considered for miRNA biomarker discovery. The use of a spike-in is the bare minimum. Although it was previously shown that the use of spike-in miRNAs in RNA isolation from serum and plasma resulted in high variability between samples, and should not be used as a normalizer for RT-qPCR analysis, our study confirmed that cel-miR-39 recovery does provide a good indication for loss of RNA during workup when comparing recovery levels from different procedures. This is especially useful for less common biofluids, like bile or preservation fluids, and, to a lesser extent, in urine, where the identification of a suitable normalizing miRNA will even be more difficult than it is in serum and plasma. Recovery of cel-miR-39, when added to serum or urine was also most optimal with the RN method. Although we did not include any method that is serum/plasma or urine specific, our data suggests that RNA, isolated with the standard, more general, miRNA isolation methods, can also provide good downstream results. This clearly shows that more general methods for RNA isolation are robust and applicable for a wide range of biofluids.

RT-qPCR is the preferred choice to measure miRNA levels in biofluids, as other methods are at present less sensitive. Other downstream platforms might require other miRNA isolation methods to obtain more reliable data. Srinivasan et al. performed an extensive analysis of ten methods in five different biofluids and analyzed the RNA samples using small RNA-seq. Their study showed that the CU method was considered the most reproducible for the analyzed bile samples, however with low complexity, suggesting further investigation was warranted [38]. Recently, Godoy et al. performed a comprehensive RNA-seq analysis, for which they also used the miRNeasy method for RNA isolation [39], suggesting that this method can be applied for various downstream applications.

Anticoagulants are amongst the most notorious inhibitors of RT-qPCR analysis. Heparin contamination, however, is not only a confounding factor in the analysis of plasma or serum, but can also play an important role in other biofluids, like urine and preservation fluids, as was previously shown [35, 36]. This phenomenon, as also shown in this study, appeared independent of the RNA isolation methods used. Co-purification of heparin occurred at a variable level, in every method we analyzed, and the use of heparin-degrading enzymes is, therefore, recommended. Detection levels of cel-miR-54, the spike-in added to the isolated RNA samples to detect any remaining RT-qPCR inhibition, were shown to be very consistent between all serum, urine and perfusate samples when heparinase I treatment was included in the RT-qPCR analysis. This suggests that other inhibitory compounds, if present in any of these three biofluids, were not co-purified or only present in very small, insignificant, concentrations. The presence of inhibitory compounds other than heparin can never be excluded as shown in RNA samples isolated from bile using method CU, where the presence of biliverdin strongly affects qPCR detection. This clearly shows that the possibility of co-purification of inhibitory components is both biofluid and isolation method dependent, and attention should be paid to their presence.

Despite the attention on RT-qPCR inhibiting compounds, our results provided evidence that there are also components present in biofluids that can actually reduce the detrimental effect of heparin on downstream applications like RT-qPCR. Where spiked-in miRNAs in a synthetic solution, such as fresh UW, showed no clear difference in kinetics between the RN and NG method, serum or urine contain compounds that affect the co-elution of heparin when the NG method was used. These varying results do emphasize the need for standard heparinase I treatment to avoid conflicting results.

Throughout this study, we focused our attention on biofluids that are related to liver transplantation and that have shown to be useful in identifying and applying miRNAs as biomarkers for complications after this surgical procedure. Although a limited set, the biofluids used in this study represent, perhaps not only the most hostile conditions to RNA, but also the most challenging fluids from which to isolate RNA. It should also be noted that we used a selection of RNA isolation methods. These are, however, among the more frequently used methods and therefore represent methods that are applied in many scientific papers. The fact that we didn’t include methods that are specific for a single biofluid was intentional as we evaluated robustness and general applicability.

## Conclusions

In this report, we compared four different methods for total RNA isolation on a diverse set of biofluids. We observed that treatment of isolated RNA with heparinase I is essential, and methods that are more broadly applicable are also suitable for biofluids for which specialized isolation methods have been developed, like serum and urine. In addition, not only heparin, but other substances, like billiverdin in bile, are also identified to inhibit RT-qPCR results, and co-purify with a subset of the methods presented in this study.

## Methods

### Sample collection and processing

Clinical samples were obtained from patients undergoing liver transplantation surgery performed at the Erasmus MC in Rotterdam, the Netherlands. Bile from liver grafts was collected and processed essentially as described previously [27]. Graft preservation fluid (perfusate) samples were obtained during the back-table procedure. Upon arrival at the operating room, grafts were flushed ex situ with University of Wisconsin solution (Viaspan, Duramed Pharm Inc., Pomona, NY), followed by a flush of human albumin solution (Albuman human albumin 40 g/l, Sanquin, The Netherlands) just prior to implantation. The effluent of this second flush was collected and processed as described by Verhoeven et al. [17]. Blood and urine were obtained within 24 h after surgery and processed immediately. Blood was collected in Vacutainer serum tubes (Becton Dickinson, Breda, The Netherlands), centrifugated at 18 °C for 10 min at 800 g to separate serum. Urine was collected as described previously [35]. Cell-free material was stored at − 20 °C until further use.

### Drugs and reagents

Heparin used in this study was laboratory grade, and in-house manufactured by the hospital pharmacy (500 IU/mL). For co-purification analysis heparin was diluted in standard saline solution (0.7%) in a serial dilution.

### RNA isolation

Total RNA was extracted using four different protocols; (RN) Qiagen miRNeasy kit (Qiagen, Venlo, The Netherlands), (QP) Qiazol (Qiagen, Venlo, The Netherlands) in combination with the precipitation carrier (dr. GenTLE, Takara, Kusatsu, Japan), (NG) NORGEN Total RNA isolation kit (Norgen biotek, Thorold, Canada), and (CU) miRCURY RNA isolation kit - biofluids (Exiqon, Vedbæk, Denmark). For QP, 2 × 100 μl of sample was lysed in 2 × 1 ml Qiazol as described by the manufacturer. After adding 200 μl chloroform, samples were centrifuged for 15 min, 12,000 x *g* at 4 °C. 500 μl of the upper, aqueous, phase of both samples was transferred to one new collection tube. RNA was precipitated by subsequent addition of 100 μl 3 M NaAc (pH 5.2), 10 μl of Dr. Gentle precipitation solution, and 1 ml iso-propanol, with vortexing after each addition. Samples were kept for 10 min at room temperature, followed by centrifugation for 10 min, 12,000 x *g* at 4 °C. Pellets were washed with 1 ml 75% ethanol, mixed by vortexing and centrifuged for 5 min, 7500 x g at 4 °C. After a second wash, pellets were dried for 10 min at room temperature and RNA was dissolved in RNAse free water. Isolation of RNA using miRNeasy, NORGEN, and miRCURY columns were executed as described in the manufacturer’s guidelines. The main properties of these isolation methods are indicated in Table 1. Step-by-step protocols can be found in the Additional File 1. In all cases, samples were spiked with 200 amol of artificial *Caenorhabditis elegans* miR-39 (cel-miR-39, Sigma Aldrich, Zwijndrecht, The Netherlands) during the lysis procedure to monitor loss during workup and 100 amol cel-miR-54 (Sigma) during cDNA synthesis to detect residual heparin contamination and other PCR inhibiting compounds co-purified with RNA.

**Table 1:** Properties of RNA isolation methods

	RN	QP	NG	CU
Sample volume (μl)	200	2 × 100	200	200
Phenol extraction	Yes	Yes	No	No
Precipitation carrier	No	Yes	No	No
Column-based	Yes	No	Yes	Yes
(Elution) volume (μl)	30	30	50	50

### Heparinase I treatment

5 μL of isolated total RNA was added to an RT reaction mixture containing 6 IU heparinase I (New England Biolabs, Ipswich, MA) and heparin degradation was obtained during the RT step for cDNA synthesis as described previously [35]. heparinase I treatment is included unless indicated otherwise.

### Reverse transcriptase and quantitative real-time polymerase chain reaction

cDNA was synthesized using the Taqman microRNA Reverse Transcription Kit (Applied Biosystems/Life technologies, Carlsbad, CA) as described previously [17], using 5 μL of isolated total RNA. cDNA was diluted to 100 μL with water and stored at − 20 °C. PCR reactions were conducted on an Applied Biosystems StepONE plus real-time PCR machine (Applied Biosystems) according to the manufacturer’s guidelines with 45 cycles of amplification. Reactions consist of 6 μL Taqman Universal PCR Master mix (Life technologies), 0.5 μL miRNA specific primer, 0.5 μL sterile milliQ water and 5 μL of diluted cDNA. The mature sequences of miRNAs analyzed, both endogenous and synthetic, are summarized in Table 2. Threshold levels for quantification of PCR results were manually set at 0.25 for all microRNA assays, and the upper Cq limit for reliable detection was set at 35 cycles. Heparinase I was included in the RT reaction unless indicated otherwise.

**Table 2:** Mature miRNA sequences and assays

miRNA	Mature sequence	Assay ID*
cel-miR-39	UCACCGGGUGUAAAUCAGCUUG	000200
cel-miR-54	UACCCGUAAUCUUCAUAAUCCGAG	001361
hsa-miR-21	UAGCUUAUCAGACUGAUGUUGA	000397
hsa-miR-30e	UGUAAACAUCCUUGACUGGAAG	002223
hsa-miR-92a	UAUUGCACUUGUCCCGGCCUGU	000431
hsa-miR-122	UGGAGUGUGACAAUGGUGUUUG	002245
hsa-miR-222	AGCUACAUCUGGCUACUGGGU	002276

*: Assay identification number as provided by ThermoFisher for the detection of individual miRNAs

### Statistical analysis

Levels of miRNAs as detected by PCR were converted to detection levels using the following equation: detection levels = 2^(−Cq)^× 10^9^. Wilcoxon matched paired tests, Mann-Whitney U tests and nonlinear regression for dose-response analyses were performed using Graphpad Prism 5.0 (Graphpad Software, San Diego, CA). *p*-values < 0.05 were considered significant.

## Supplementary Information

**Additional file 1.** Step-by-step protocols for RNA isolation methods used throughout this manuscript.

**Additional file 2.** Combining the most efficient isolation method with the one least sensitive for co-isolation of heparin does not improve overall outcome.

## Data Availability

Data used and/or analyzed in this study are available from the corresponding author upon reasonable request.

## Abbreviations

miR/miRNA: microRNA
RT-qPCR: reverse transcription quantitative real-time PCR
cel: *Caenorhabditis elegans*
hsa: *Homo sapiens*
QP: Classic phenol-chloroform extraction with Dr.GenTLE as a precipitation carrier
RN: miRNeasy mini kit
NG: NORGEN total RNA purification kit
CU: miRCURY RNA Isolation Kit – Biofluids
