# Supplementary material for: Evaluation of RNA isolation methods for microRNA quantification in a range of clinical biofluids
Source: BMC Biotechnol. 2021 Aug 6;21:48. doi: 10.1186/s12896-021-00706-6 (PMC8344161; doi:10.1186/s12896-021-00706-6)
Supplement: Supplementary file 1 — Additional file 1. Step-by-step protocols for RNA isolation methods used throughout this manuscript. [file 12896_2021_706_MOESM1_ESM.docx]

**RNA isolated methods**

Step-by-step protocols

Qiagen miRNeasy kit (RN)

1. Add 1400 µl QIAzol Lysis Reagent to 200 µl sample in a 2 ml tube and disrupt and homogenize sample by shaking for 30’’.

2. Place tube containing homogenate at room temperature for 5 min.

3. Add 20 µl 10 pM cel-miR- 39

4. Add 280 µl chloroform and cap tube securely. Shake vigorously for 15 sec.

5. Place tube at room temperature for 2-3 min.

6. Centrifuge for 15 min. at 12,000 x g at 4°C.

7. Transfer upper aqueous phase (800 µl) to new 2 ml collection tube. Avoid pipetting the (white) interface. Add 1.5 volumes of 100% ethanol (i.e. 1.2 ml) and mix thoroughly by pipetting.

8. Pipet up 650 µl of sample, including any precipitate, onto an RNeasy Mini column in 2 ml collection tube. Close lid, centrifuge at 10.000 rpm for 15 sec. at room temperature (15-25°C). Discard flow-through.

9. Repeat step 8 until all of the sample has been loaded on the cloumn.

10. Add 700 µl Buffer RWT to the RNeasy Mini column. Close lid, centrifuge for 15 sec. at 10.000 rpm. Discard flow-through.

11. Pipet 500 µl Buffer RPE onto RNeasy Mini column. Close lid, centrifuge for 15 sec. at 10.000 rpm. Discard flow-through.

12. Add 500 µl Buffer RPE to RNeasy mini column. Close lid, centrifuge for 2 min. at 10.000 rpm.

13. Dry the outside of the column with a clean tissue.

14. Transfer RNeasy Mini column to new 1.5 ml collection tube from the box. Pipet 30 µl RNase-free water directly onto RNeasy Mini column membrane. Close lid, centrifuge for 1 min. at 10.000 rpm to elute.

15. Re-apply the 30 µl eluate to the column and spin again to completely elute

16. Store at -80°C

Qiazol and Dr. Gentle Total RNA isolation (QP)

1. Add 1 ml QIAzol Lysis Reagent to 100 µl sample in a 2 ml tube and disrupt and homogenize sample by shaking for 30’. Do this in duplicate! (i.e. You have 2 tubes each with 100 µl per sample)

2. Place tubes containing homogenate at room temperature for 5 min.

3. Meanwhile, add 10 µl 10 pM cel-miR-39 to each tube

4. Add 200 µl chloroform per ml Qiazol used and cap tube securely. Shake vigorously for 15 sec.

5. Place tube at room temperature for 2-3 min.

6. Centrifuge for 15 min. at 12,000 x g (=rcf) at 4°C.

7. Transfer upper aqueous phase of the same sample (2x 500 µl) to new 2 ml collection tube.

8. Add 100 µl 3M NaAc (pH 5.2) (i.e. 1/10 V of the total aqueous phase) and short vortex

9. Add 1 µl of Dr. Gentle precipitation solution per 100 µl upper phase (= 10 µl) and short vortex.

10. Add 1 ml (0.5 volume of iso-propanol per ml Qiazol used = 2 ml) and mix thoroughly by pipetting.

11. Incubate 10 min at RT.

12. Centrifuge for 10 min. at 12,000 x g at 4°C

13. Carefully remove the supernatant with a vacuum pump. Use RNAse free tips!

14. Wash the “pellet” with 1 ml of 75% ethanol. Vortex

15. Centrifuge 5’, 7500 x g at 4°C.

16. Remove the supernatant and repeat step 14-15

17. Dry for 10’ at RT

18. Dissolve in 30 µl RNAse free water

19. Store at -80°C

NORGEN total RNA purification kit (NG)

1. Add 10 µl β-mercapto-ethanol per ml RL buffer just prior to use

2. Add 0.7ml RL buffer (containing β-mercapto) to 0.2 ml sample in a 2 ml tube.

3. Vortex ~15 seconds

4. Add 20 µl 10 pM cel-miR-39

5. Add 0.4 ml 100% ethanol

6. Vortex ~10 seconds

7. Place each column in a collection tube without a lid

8. Load 650 µL sample onto the column in a collection tube

9. Centrifuge 1’ 8000 RPM at RT

10. Discard the flow-through

11. Repeat steps 8-10 until the complete sample is loaded

12. Add 400 µl Wash solution A

13. Centrifuge 1 min at 13200 RPM at RT and discard flow-through

14. Repeat steps 12-13 2x (=3 times washing in total)

15. Spin 2’ at 13200 RPM at RT to dry column

16. Dry the outside of the column with a clean tissue

17. Place the column in a fresh 1.5 ml tube

18. Add 50 µl Elution solution A

19. Spin 2 min at 2000 RPM directly followed by a…

20. Spin 1 min at 13200 RPM

21. Check eluted volume. If<50 µl then repeat step 18.

22. Store at -80°C

mirCURY RNA Isolation Kit - Biofluids (CU)

1. Add 60 µl Lysis solution to 0.2 ml sample.

2. Vortex ~5 seconds

3. Place tube containing homogenate at room temperature for 3 min.

4. add 20 µl of 10 pM cel-miR-39

5. Add 22 µl Protein precipitation solution

6. Vortex ~5 seconds

7. Place tube at room temperature for 1 min.

8. Centrifuge for 3 min. at 11000 x g at RT.

9. Transfer clear supernatant to a new 2 ml tube.

10. Add 270 µl isopropanol

11. Vortex ~5 seconds

12. Load sample onto the column in a fresh collection tube

13. Incubate 2’ at RT

14. Centrifuge 30” 11000 x g at RT

15. Discard the flow-through

16. Add 100 µl Wash solution 1

17. Centrifuge 30” 11000 x g at RT and discard flow-through

18. Add 700 µl Wash solution 2

19. Centrifuge 30” 11000 x g at RT and discard flow-through

20. Add 250 µl Wash solution 2

21. Centrifuge 2 min, 11000 x g at RT

22. Place the column in a fresh 1.5 ml tube and add 25 µl RNAse-free water direct on the column

23. Incubate at RT for 1 min.

24. Centrifuge for 1 min. at 11000 x g at RT.

25. Repeat step 22-24

26. Store at -80°C
