# Supplementary material for: Evaluation of RNA isolation methods for microRNA quantification in a range of clinical biofluids
Source: BMC Biotechnol. 2021 Aug 6;21:48. doi: 10.1186/s12896-021-00706-6 (PMC8344161; doi:10.1186/s12896-021-00706-6)
Supplement: Supplementary file 2 — Additional file 2. Combining the most efficient isolation method with the one least sensitive for co-isolation of heparin does not improve overall outcome. [file 12896_2021_706_MOESM2_ESM.docx]

**
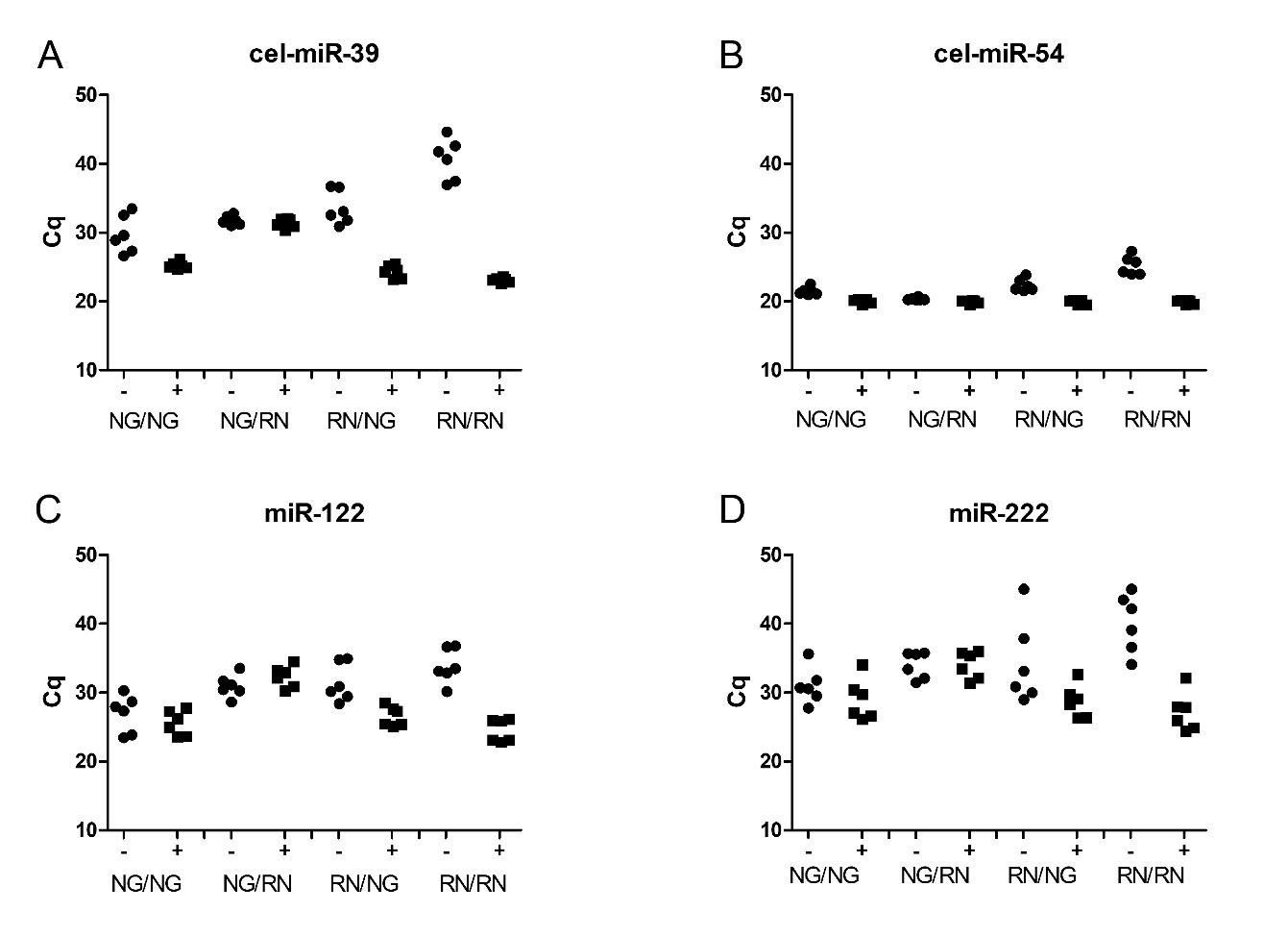
**

**Combining the most efficient isolation method with the one least sensitive for co-isolation of heparin does not improve overall outcome.** Quantification levels (Cq) of synthetic miRNAs Cel-miR-39 (A), and Cel-miR-54 (B), and of endogenous miR-122 (C), and miR-222 (D) determined in preservation fluid PF2 with established contamination with heparin of 6 donor livers grafts prior to transplantation (1) . NG/..; Norgen kit lysis and denaturation, ../NG; Norgen kit column isolation, RN/..; Qiazol lysis, ../RN; miRNEAsy column isolation. The RN/RN method remains the most efficient, but with the highest levels of co-purified heparin. NG/NG remains the least affected by heparin contamination, but neither NG/RN nor RG/RN combined into a more superior method.

1. Selten JW, Verhoeven CJ, Heedfeld V, Roest HP, de Jonge J, Pirenne J, et al. The release of microrna-122 during liver preservation is associated with early allograft dysfunction and graft survival after transplantation. Liver Transpl 2017;23:946-56.
